# Towards automated evaluation of left atrial transit time

**DOI:** 10.1186/1532-429X-16-S1-P36

**Published:** 2014-01-16

**Authors:** Yongpeng Tang, Michael Passick, Jie J Cao, Yi Wang

**Affiliations:** 1 Research and Education, St. Francis Hospital, Roslyn, New York, USA

## Background

Left atrial transit time (LATT) has potential to approximate left ventricular end diastolic pressure noninvasively. Small artifacts are often present in the down-slope portion of the time-intensity curve most likely due to poor SNR resulting from relatively low contrast dosing. While easily circumvented by experienced operator the artifact presents a challenge to automated detection of area under the curve (AUC) which is essential in the determination of LATT. To minimize user interface we sought to develop a filtering algorithm in the post processing and compared automated AUC detection with operator directed AUC assessment.

## Methods

**Figure 1:**
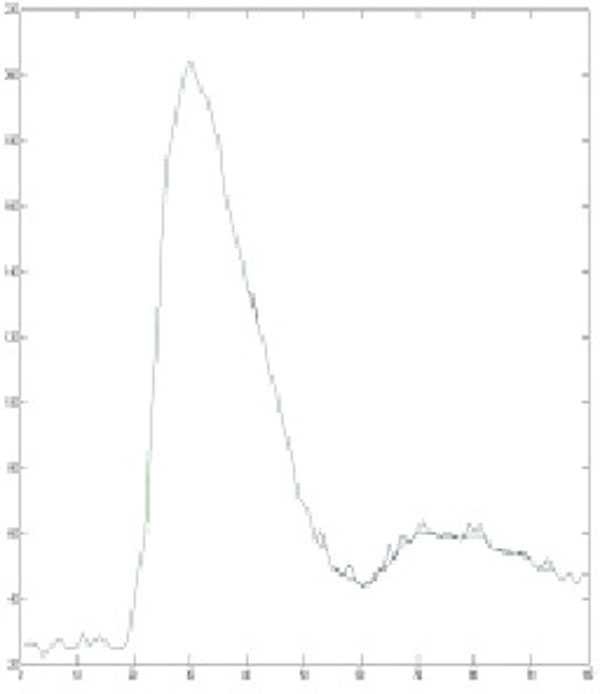
**Shows an example of original and filtered curves**.

Thirty seven patients (age: 54.1 ± 15.3, 9 females) were studied. All subjects underwent first pass perfusion using the SSFP saturation recovery sequence with ECG gating and breath-hold during gadolinium bolus injection at 0.01 mmol/kg. A time-intensity curve was generated by tracing and plotting the blood signals in the left atrium including all image phases and processed in a custom Matlab program with and without the filtering algorithm. The unfiltered and filtered AUC were compared to the operator directed assessment. The example in Figure 1 shows a time-intensity curve from unfiltered data. Without filtering the AUC ended at phase 52 due to an artifact, while a filtered program detected AUC up to phase 60 which coincided with the AUC assessed by an experienced operator.

## Results

**Figure 2:**
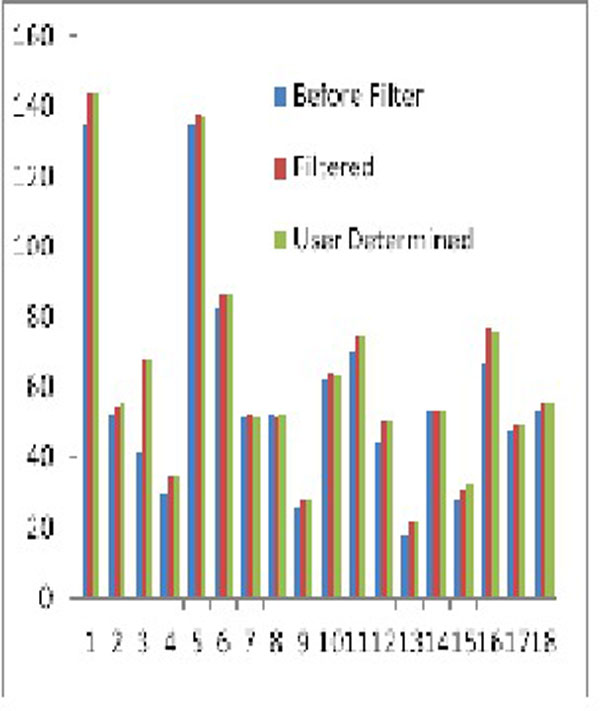
**Shows the AUC comparison in all 18 cases**.

**Figure 3:**
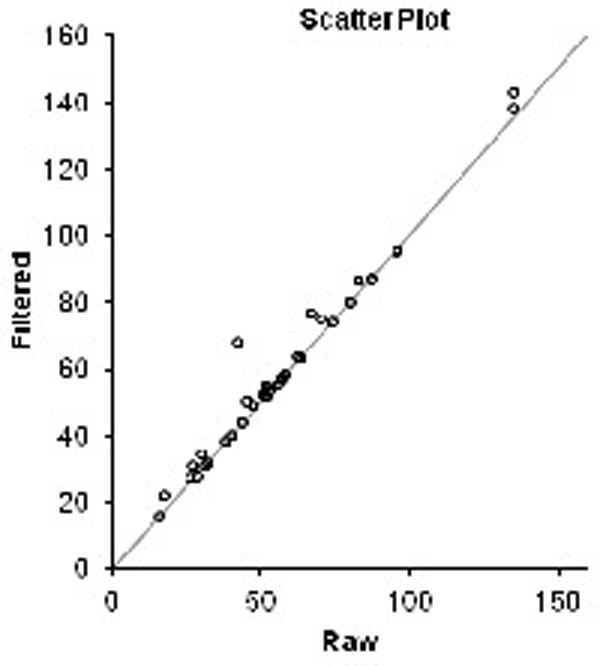
**(left) Shows the Bland Altman plot of filtered versus unfiltered AUC**.

**Figure 4:**
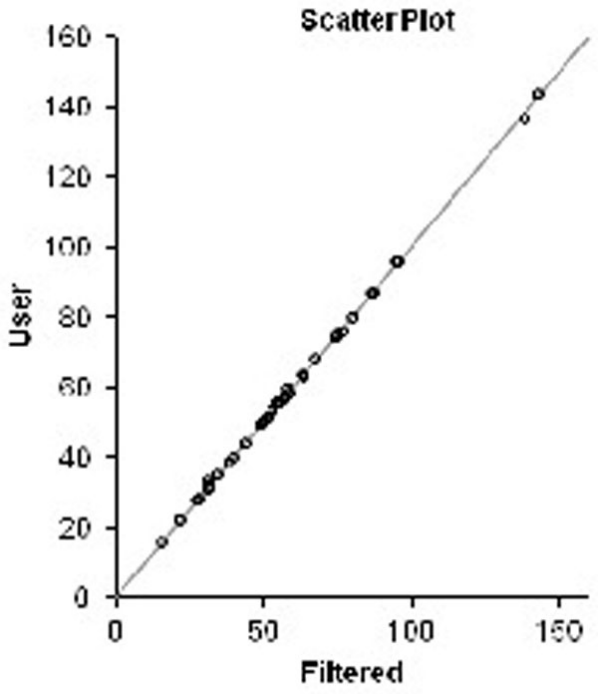
**(right) Shows the Bland Altman plot of filtered versus operator directed AUC**.

Of the 37 cases analyzed, artifacts were present in 18 cases. The filtering algorithm was successful at detecting and removing artifacts in all 18 cases. As a result, the filtered AUC was much closer to the operator directed AUC shown (in green and in red, respectively) in Figure 2 than unfiltered AUC (in blue) was. Bland-Altman analysis demonstrated a much improved agreement between filtered and operator directed AUC detection (Figure 4) than filtered and unfiltered AUC (Figure 3).

## Conclusions

Artifact is common in time-intensity curve of the LA blood signal. A filtering algorithm in the post process is promising to successfully remove the artifact allowing automated assessment of AUC.

## Funding

None.

